# A comprehensive population-based study comparing the phenotype and genotype in a pretherapeutic screen of dihydropyrimidine dehydrogenase deficiency

**DOI:** 10.1038/s41416-020-0962-z

**Published:** 2020-06-29

**Authors:** Nicolas Pallet, Salma Hamdane, Simon Garinet, Hélène Blons, Aziz Zaanan, Elena Paillaud, Julien Taieb, Olivier Laprevote, Marie-Anne Loriot, Céline Narjoz

**Affiliations:** 1 Department of Clinical Chemistry, Hôpital Européen Georges Pompidou, Assistance Publique Hôpitaux de Paris, University of Paris, Paris, France; 2 Department of Gastroenterology and Gastrointestinal Oncology, Hôpital Européen Georges Pompidou, Assistance Publique Hôpitaux de Paris, University of Paris, Paris, France; 3 Geriatric Oncology Unit, Paris Cancer Institute CARPEM, Hôpital Européen Georges Pompidou, Paris, France; 4 Université Paris Est Creteil, INSERM, IMRB, F-94010 Creteil, France

**Keywords:** Cancer genomics, Biochemistry

## Abstract

**Background:**

Pretherapeutic screening for dihydropyrimidine dehydrogenase (DPD) deficiency is recommended or required prior to the administration of fluoropyrimidine-based chemotherapy. However, the best strategy to identify DPD-deficient patients remains elusive.

**Methods:**

Among a nationwide cohort of 5886 phenotyped patients with cancer who were screened for DPD deficiency over a 3 years period, we assessed the characteristics of both DPD phenotypes and *DPYD* genotypes in a subgroup of 3680 patients who had completed the two tests. The extent to which defective allelic variants of *DPYD* predict DPD activity as estimated by the plasma concentrations of uracil [U] and its product dihydrouracil [UH_2_] was evaluated.

**Results:**

When [U] was used to monitor DPD activity, 6.8% of the patients were classified as having DPD deficiency ([U] > 16 ng/ml), while the [UH_2_]:[U] ratio identified 11.5% of the patients as having DPD deficiency (UH_2_]:[U] < 10). [U] classified two patients (0.05%) with complete DPD deficiency (> 150 ng/ml), and [UH_2_]:[U] < 1 identified three patients (0.08%) with a complete DPD deficiency. A defective *DPYD* variant was present in 4.5% of the patients, and two patients (0.05%) carrying 2 defective variants of *DPYD* were predicted to have low metabolism. The mutation status of *DPYD* displayed a very low positive predictive value in identifying individuals with DPD deficiency, although a higher predictive value was observed when [UH_2_]:[U] was used to measure DPD activity. Whole exon sequencing of the *DPYD* gene in 111 patients with DPD deficiency and a “wild-type” genotype (based on the four most common variants) identified seven heterozygous carriers of a defective allelic variant.

**Conclusions:**

Frequent genetic *DPYD* variants have low performances in predicting partial DPD deficiency when evaluated by [U] alone, and [UH_2_]:[U] might better reflect the impact of genetic variants on DPD activity. A clinical trial comparing toxicity rates after dose adjustment according to the results of genotyping or phenotyping testing to detect DPD deficiency will provide critical information on the best strategy to identify DPD deficiency.

## Background

Chemotherapy with fluoropyrimidine drugs, such as fluorouracil, capecitabine and tegafur, is the standard treatment for many types of advanced cancer and is used by millions of patients with cancer worldwide.^1^ However, 15–30% of patients will experience severe treatment-related toxicity, which is lethal in 0.5–1% of patients.^2–4^ Capecitabine and tegafur are metabolised to fluorouracil, an anti-metabolite and pyrimidine analog that plays a pivotal role in the occurrence of this toxicity. The most well-known biochemical cause of intolerance to fluoropyrimidines is a deficiency in the catabolic enzyme dihydropyrimidine dehydrogenase (DPD).^5^ Patients with partial or complete DPD deficiency have a reduced capacity to degrade fluorouracil and are at risk of developing severe fluoropyrimidine-associated toxicity.

Genetic polymorphisms in *DPYD*, the gene encoding DPD, predict fluoropyrimidine-associated toxicity.^1,6–9^ Pre-treatment screening for the most clinically relevant defective variants, c.1679T>G, c.1905 + 1G>A, c.2846A>T, and Haplotype B3 (c.1236G>A or c.1129–5923C>G), and a dose adjustment according to the *DPYD* genotype improves the safety of chemotherapy regimens based on fluorouracil.^10,11^ International recommendations provide indications for drug-related genetic tests and *DPYD* genotype-guided dosing to improve their integration in routine clinical practice.^6,12^ The U.S. Food and Drug Administration and the Health Candida Santé Canada have added statements to the drug labels for fluorouracil and capecitabine that warn against use in patients with DPD deficiency, and genotype-guided prescribing recommendations for fluorouracil, capecitabine, and tegafur are also available from the Dutch Pharmacogenetics Working Group.^12^ The Clinical Pharmacogenetics Implementation Consortium (CPIC) has also proposed guidelines for the genotype-guided dosing of fluoropyrimidines.^6^

Another approach to identify DPD-deficient patients is to assess DPD enzyme activity by determining the plasma concentrations of uracil ([U]), the endogenous substrate for DPD, or its product dihydrouracil (UH_2_) to calculate the [UH_2_]:[U] ratio.^1^ [U] and the [UH_2_]:[U] ratios are highly correlated with DPD activity in peripheral blood mononuclear cells (PBMCs).^1^ Although debate exists regarding whether [U] or the [UH_2_]:[U] ratio correlates well with the clearance of fluorouracil,^13,14^ numerous studies have identified a relationship between fluoropyrimidine-induced toxicity and a DPD phenotype characterised by a high [U] or a low [UH_2_]:[U] ratio.^1,13–15^ Measuring the DPD phenotype has the potential to improve the performance of the prechemotherapy tests designed to identify patients at risk of fluoropyrimidine-associated toxicity.^1,5,7,14,16^ Additionally, dose individualisation in patients with DPD deficiency as evaluated by measuring [UH_2_] and [U] may improve the safety of these patients.^17–19^ A fast and reliable quantitative analysis of the DPD phenotype is performed with an accurate, precise, robust and sensitive liquid chromatography tandem mass spectrometry assay.^20,21^ Since December 2018 in France, prior to the initiation of treatment with fluorouracil or capecitabine, a search for DPD deficiency must be conducted by determining the pretherapeutic [U].^22^ Prescribers of fluoropyrimidines must mention on the prescription that the results of plasma [U] have been considered, and the pharmacist must ensure the presence of this statement before dispensation. The French guidelines do not include the [UH_2_]:[U] ratio as an indicator for the DPD metabolisation status. Thus, the mean and range of the pretherapeutic endogenous [UH_2_]:[U] ratio varies widely between studies, and the extent to which the [UH_2_]:[U] ratio correlates with fluorouracil plasma concentrations remains a subject of discussion.^13,14^ Indeed, [U] might be superior to the [UH_2_]:[U] ratio as a predictor of severe toxicity, and pre-treatment [U] concentrations > 16 ng/ml are strongly associated with global severe toxicity, with the risk of severe toxicity increasing proportionally with increasing [U] values.^1,14^

To date, the most appropriate strategy of screening for DPD deficiency is a highly debated topic, and data from large-scale studies designed to establish the respective performances of phenotyping, genotyping and combined approaches are lacking. An important and yet unresolved issue is to what extent *DPYD* genotypes reflect pre-treatment DPD activity estimated by measuring [U] or [UH_2_]:[U] ratio.^1^ We took advantage of the extensive experience of the pharmacogenetics unit of a French university hospital in testing for pretherapeutic screening of DPD deficiency to address this issue.^21^ We performed a cross-sectional observational study that comprehensively describes the DPD phenotypic characteristics among 3680 patients. Complementary genetic testing has been performed for common *DPYD* variants in all patients and rare variants in selected cases, and the relationship between the *DPYD* genotype and DPD phenotype has been analysed.

## Methods

We retrospectively reviewed the laboratory database of the Hôpital Européen Georges Pompidou (Assistance Publique—Hôpitaux de Paris). All patients referred to the pharmacogenetics unit (2016–2019) for pre-treatment testing for DPD deficiency were considered (Fig. S1). Screening for DPD deficiency was performed at the discretion of the physician. Genotyping for the four common genetic variants of *DPYD* (c.1679T>G, c.1905 + 1G>A, c.2846A>T, and c.1129–5923C>G (Haplotype B3)) was performed in 5513 patients and phenotyping by measuring [U] and [UH_2_]:[U] was performed in 5886 patients. The distribution of the [U] values of the 5886 patients is shown in Fig. S2. Overall, 3680 patients were screened for DPD deficiency by both genotyping and phenotyping (Fig. S1). Whole-exome sequencing of the *DPYD* gene was performed in 111 individuals with a wild-type genotype, and DPD deficiency was defined as [U] > 16 ng/ml^7,14^ and/or [UH_2_]:[U] < 10 ^14^ (see the “Phenotyping” section). This cross-sectional study is reported according the STROBE statement.

### Phenotyping

[U] and [UH_2_] were measured using a Waters Acquity® TQD LC®/MS/MS System consisting of an Acquity® ultra high-performance liquid chromatography system (Waters; Milford, MA) coupled with an Acquity® triple-quadrupole tandem mass spectrometer with an electrospray ionisation interface. Complete validation was performed according to the Food and Drug Administration guidelines on bioanalytical method validation. All data were acquired and processed using MassLynx™ 4.1 software with the QuanLynx™ program. The detailed technical protocol is described elsewhere.^21^ Based on the published data available to define a uracil threshold discriminating normal and deficient activity, a clinically relevant increase in the risk of severe toxicity occurs when [U] is >16 ng/ml.^1,7,11,14^ Therefore, the following cut-off values were used throughout the study: normal activity if [U] < 16 ng/ml and partially deficient activity if 16 ng/ml < [U] < 150 ng/mL.^1,7^ Completely deficient activity was defined as [U] > 150 ng/mL after a survey of 38,862 patients from the French laboratories performing DPD phenotyping (unpublished results). Thus, a [U] threshold value of 150 ng/ml was proposed as a consensus by experts to identify complete deficits. The cut-of value of [UH_2_]:[U] ratio to discriminate DPD deficiency may vary according to studies, mostly due to technical specificities of HPLC-UV^16,18,23^ or HPLC-MS^13,21,24^ to measure [U] and [UH_2_]. In the present study, a [UH_2_]:[U] ratio cut-off of < 10 has been chosen because it has been demonstrated that it was a good predictor of toxicity.^14^ Thus, partial DPD deficiency was defined when [UH_2_]:[U] < 10, and complete DPD deficiency was defined as [UH_2_]:[U] < 1.^14,17,18,23^

### Genotyping

Genotyping was restricted to patients who consented to genetic analyses. Conventional PCR-based assays were used to detect the four *DPYD* polymorphisms with strong evidence supporting defective function (c.1679T>G, c.1905 + 1G>A, c.2846A>T, and c.1129–5923C>G) using the TaqMan® DME Assay (Applied Biosystems, France) for allelic discrimination. Based on the genotype result, an activity score is calculated to predict whether the patient has normal, intermediate or low metabolism.^6^ An activity score of 2 indicates that a patient has two fully functional alleles (activity score: 1 + 1), predicting normal metabolism. An activity score of 1 to 1.5 signifies that a patient is a carrier of one fully functional allele (=1) and one allele with decreased activity (=0.5), 2 alleles with decreased activity (0.5 + 0.5), or one fully functional allele (=1) and one non-functional allele (=0), predicting intermediate metabolism. An activity score of 0.5 or 0 signifies that a patient has 1 non-functional allele (=0) and one allele with decreased activity (=0.5) or 2 non-functional alleles (0), predicting low metabolism. Among the four common genetic variants detected by genotyping, c.1679T>G and c.1905 + 1G>A are considered “non-functional” alleles (activity score 0), and c.2846A>T and c.1129–5923C>G are considered “decreased activity” alleles (activity score 0.5).

### Next-generation sequencing

Samples were characterised for molecular alterations using targeted Next-Generation Sequencing (Ion AmpliSeq™ Custom 5FUIRI IAD68279, Life Technologies™, Carlsbad, CA). Briefly, the multiplex barcoded libraries were generated from 10 ng of DNA according to the manufacturer’s recommendations (Ion ampliseq library kit V2) and normalised using the Ion Library Equalizer™ Kit. The pooled libraries (max 96) were processed with the Ion Chef™ System for template preparation and chip loading (Ion PI HI-Q Chef Kit, Ion PI Chip V3), and sequenced using the Ion Proton™ System (Life Technologies™). A custom Ion Torrent panel has been designed with the Ampliseq Designer software using human Hg19 as reference. Primers were divided into two pools. The 23 exons of the *DPYD* gene, 50 bp of exon flanking regions, the 3′UTR region and a part of 5′UTR (300 bp upstream transcription initiation) were sequenced. The FASTQ sequencing data were aligned to the human genome (hg19) and processed using Ion Torrent Suite V5.0.4.0. This package included the Torrent Variant Caller V5.0.4.0 using the built-in Germ-line settings optimised for high-frequency settings. All variants were checked in the genome browser Alamut® Visual (Interactive Biosoftware, Rouen, France).

### Statistical analyses

Data are described as the means ± standard deviations, or *n* (%). Since [U] and [UH_2_]:[U] values were not normally distributed (Fig. S3), nonparametric Mann–Whitney and Kruskal–Wallis tests were performed. Proportions were compared using the Chi^2^ test. Spearman’s correlation coefficients were calculated to assess correlations between variables. The maximum value of the Youden index (sensitivity + specificity − 100) was calculated to identify the optimal cut-off points for [U] and [UH_2_]:[U] values. Two-sided *p*-values < 0.05 were considered significant. Statistical analyses were performed using Prism^®^ 5.0 (GraphPad, CA, USA) and JMP (SAS) software.

## Results

### DPD phenotyping by measuring plasma U and UH_2_ concentrations

**Fig. 1:**
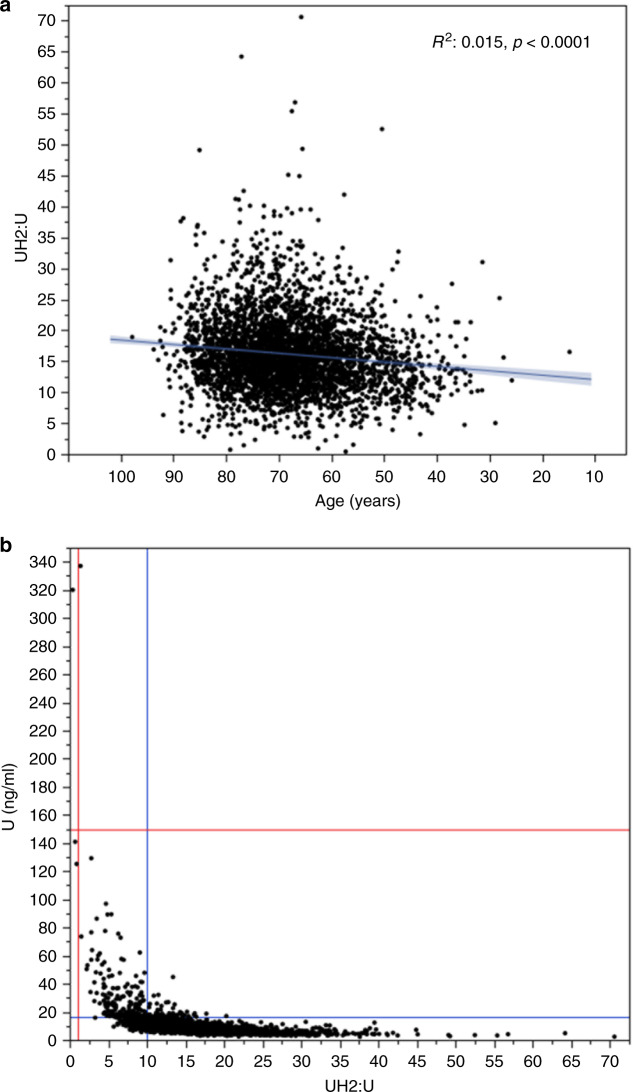
Characteristics of DPD activity. **a** Correlation between [UH_2_]:[U] and patient age. **b** Correlation between [U] and [UH_2_]:[U]. Horizontal lines represent the threshold [U] level for partial deficiency (blue) and complete deficiency (red), and vertical dashed lines represent the threshold [UH_2_]:[U] for partial deficiency (blue) and complete deficiency (red).

Mean patients age was 64.5 ± 13 years, and the sex ratio (M/F) was 41%. A negative correlation was observed between patient age and [UH_2_]:[U] (but not [U]) values, suggesting that DPD activity may decrease with age (Fig. 1a). In addition, [U] (and not [UH_2_]:[U]) values were slightly higher in men than in women (10 ± 10.9 ng/ml vs. 9.4 ± 10.1 ng/ml, *p* = 0.001, Mann–Whitney test), but the clinical relevance of this difference is questionable. Because pre-analytical conditions are critical for [U] or [UH2]:[U] measurements, and may vary according to centres that collect blood samples, we checked that there was not centre effect *i.e*. that there was no significant difference in [U] or [UH2]:[U] values from one health centre to another (Kruskal–Wallis *p*-values 0.8 for [U] and 0.18 for [UH_2_]:[U]) (data not shown).

[U] values identified 249 patients (6.8%) with partial DPD deficiency and two patients (0.05%) with complete DPD deficiency. The [UH_2_]:[U] ratio classified 423 patients (11.5%) with partial DPD deficiency, and 3 (0.08%) with complete DPD deficiency (Fig. 1b). Overall, [U] and [UH_2_]:[U] values identified 468 patients with DPD deficiency, but these parameters were in agreement in only 209 (44%) of the patients (Fig. 1b and Table 1). Indeed, 217 (5.8%) patients presented [UH_2_]:[U] < 10 and [U] > 16 ng/ml, and 42 (1.1%) presented [UH_2_]:[U] > 10 and [U] < 16 ng/ml (Table 1). The [UH_2_]:[U] level below which [U] values were all > 16 ng/ml was 5, and the [U] level above which [UH_2_]:[U] values were all < 10 was 50 ng/ml (Fig. 1b), suggesting that a better agreement between [UH_2_]:[U] and [U] values to identify DPD deficiency would require the use of more restrictive thresholds. Based on these results, the current cut-off values for [U] and [UH_2_]:[U] do not identify DPD deficiency in an equivalent manner, and a [UH_2_]:[U] ratio < 10 yields a higher proportion of individuals classified with partial DPD deficiency than [U] levels > 16 ng/ml.

**Table 1:** Congruence between [U] and [UH_2_]:[U] values for DPD deficiency.

	[U] < 16 ng/ml	[U] > 16 ng/ml
[UH_2_]:[U] < 10	217 (5.8%)	209 (5.6%)
[UH_2_]:[U] > 10	3212 (87.2)	42 (1.1%)

### Prediction of DPD phenotype by the *DPYD* genotype

**Fig. 2:**
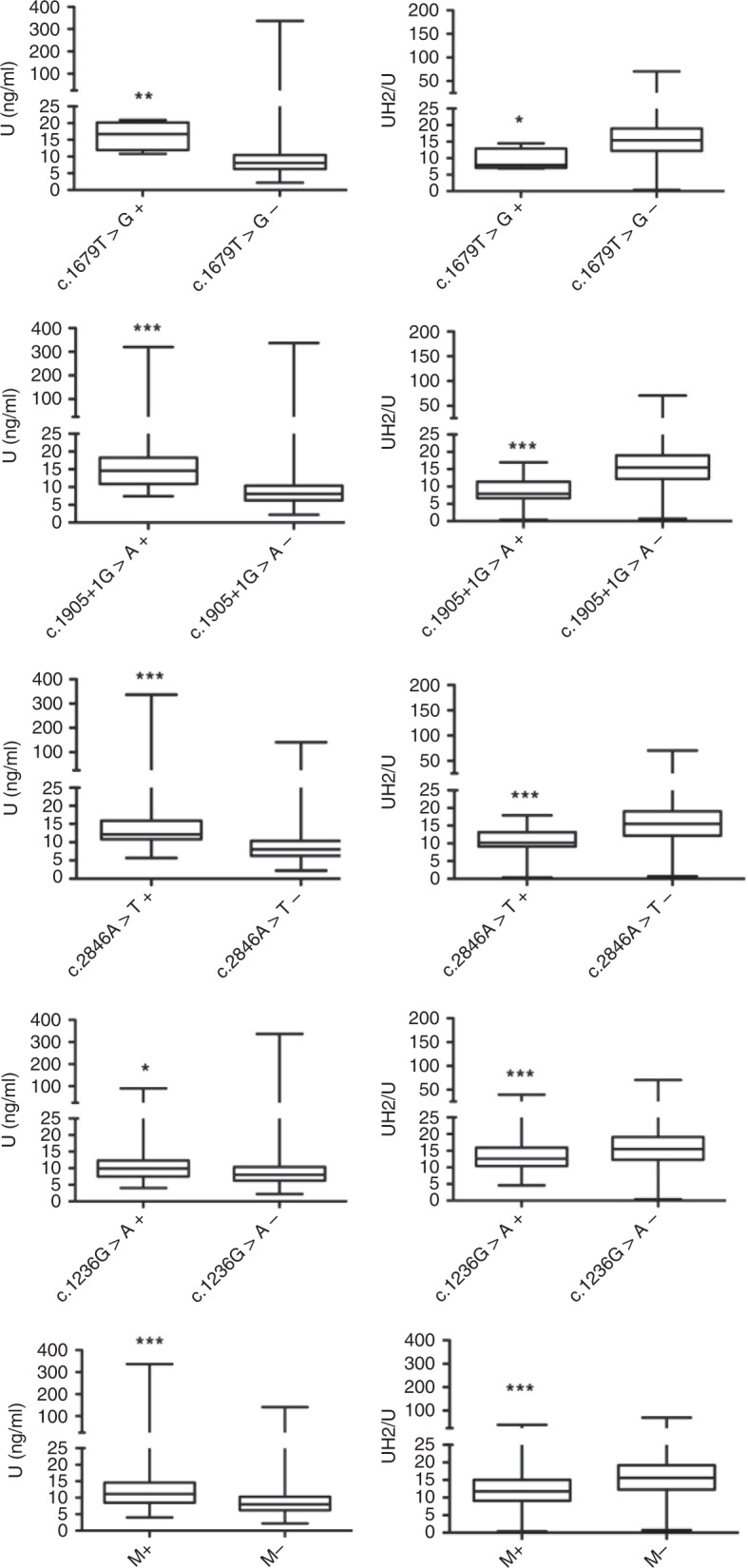
Distribution of [U] values according to the mutation status of the patients. Box and whisker plots (the ends of the box are the upper and lower quartiles, the median is marked by a vertical line inside the box, the whiskers are the two lines outside the box that extend to the highest and lowest observations) of [U] and [UH_2_]:[U] according to the presence of one of the most well-known genetic variations in the *DPYD* gene. Distributions were compared using the Wilcoxon test. *p < 0.05, **p < 0.01 and ***p < 0.001.

One hundred sixty-six (166) (4.5%) patients were carriers of one defective *DPYD* variant (Table 2), and these patients were predicted to have intermediate metabolism according to the activity score of *DPYD* alleles (1 or 1.5) (see the “Methods”).^6^ The presence of a mutation in the *DPYD* gene has a low positive predictive value (PPV) to identify individuals with partial DPD deficiency (Table 3), but the PPV of a *DPYD* mutation is higher when [UH_2_]:[U] is used to evaluate DPD activity rather than [U] (PPV 33% vs. 16%, respectively). When DPD deficiency was considered only if [U] > 16 ng/ml and [UH2]:[U] < 10, the PPV of a *DPYD* mutation slightly increased to 37%. Reflecting the poor predictive capacities of *DPYD* mutations, considerable overlap was observed in the distribution of [U] and [UH_2_]:[U] values between each group of patients according to the *DPYD* mutation status (Fig. 2). However, the negative predictive value (NPV) of *DPYD* genotyping averaged 90% for [U] or [UH_2_]:[U] (Table 3). Receiver operating characteristic (ROC) curves (Fig. S4) generated cut-off values for [U] and [UH_2_]:[U] that would assign the presence of a defective variant of *DPYD* to the optimal prediction of DPD activity: 10.5 ng/ml for [U] (PPV, 57% and NPV, 76%) and 13.7 for [UH_2_]:[U] (PPV, 69%, NPV, 64%). Together, these results indicate that the presence of a defective *DPYD* allelic variant is a poor predictor of DPD deficiency and that the predictive value further decreases when [U] is used instead of [UH_2_]:[U].

**Table 2:** Prevalence of the four most frequent *DPYD* genetic variants in the cohort of 3680 patients with cancer who were tested for DPD deficiency.

DPYD Allele	rsID	Nucleotide change	Activity score	Mutations	Allele frequency
*13	rs55886062	c.1679T>G	0 (No function)	4	0,0005
*2A	rs3918290	c.1905 + 1G>A	0 (No function)	25	0,003
	rs67376798	c.2846A>T	0.5 (Decreased function)	34	0,004
HapB3	rs56038477	c.1129–5923C>G	0.5 (Decreased function)	109	0,02

**Table 3:** Diagnostic performance of the *DPYD* variants assessed in predicting DPD enzymatic deficiency.

A					
[U] > 16 ng/ml	Allele functional status	% Sensitivity [95% CI]	% Specificity [95% CI]	% PPV [95% CI]	% NPV [95% CI]
c.1679T>G	No function	0.8 [0.09-2]	99 [99.7-99.9]	50 [7–93]	93 [92–94]
c.1905 + 1G>A	No function	3.5 [1.6–6.7]	99.5 [99.2–99.7]	36 [18–57]	93 [92–94]
c.2846 A>T	Decreased function	2.7 [1.1–5.6]	99 [98–99.5]	20.5 [8–37]	93 [92–94]
c.1129–5923C>G	Decreased function	4.3 [2.2–7.7]	97 [97–98]	10 [5–17]	93 [92–94]
Any of the 4 mutations	No or decreased function	10.7 [7–15]	95 [95–96]	16 [11–22]	93 [92–94]

B					
[UH_2_]/[U] < 10	Allele functional status	% Sensitivity [95% CI]	% Specificity [95% CI]	% PPV [95% CI]	% NPV [95% CI]
c.1679T>G	No function	0.7 [0.15–0.2]	99 [99.7–99.9]	75 [19–99]	88 [87–89]
c.1905 + 1G>A	No function	4 [2–6]	99.7 [99.5–99.8]	68 [46–85]	88 [87–89]
c.2846 A>T	Decreased function	3.7 [2–6]	99.4 [99.1–99.6]	47 [29–64]	88 [87–89]
c.1129–5923C>G	Decreased function	5 [4–8]	97 [96–98]	22 [14–30]	88 [87–89]
Any of the 4 mutations	No or decreased function	13.3 [10–17]	96 [95–97]	33 [26–41]	89 [88–90]

### Prediction of complete DPD deficiency by *DPYD* genotyping

None of the 3680 patients who were tested for the 4 *DPYD* common variants associated with impaired enzyme activity were homozygous carriers of these polymorphisms. Two patients (0.05%) carried compound heterozygous mutations, c.1905 + 1G>A plus c.2846A>T and c.1905 + 1G>A plus c.1129–5923C>G, and were predicted to have low metabolism according to an activity score of 0.5.^6^ However, only the patient carrying the c.1905 + 1G>A plus c.2846 A>T mutations displayed a complete loss of DPD activity ([U] = 320 ng/ml and [UH_2_]:[U] = 0.4), while the patient carrying the c.1905 + 1 G>A plus c.1129–5923 C>G variant alleles had normal [U] and [UH_2_]:[U] values (Table 4). The second patient with completely deficient DPD activity based on [U] values ([U] = 337) was predicted to have intermediate metabolism by *DPYD* genotyping (carrier of the c.2846A>T variant, activity score of 1.5), but the high level of [UH_2_] (479 ng/ml) suggested inadequate sample storage that may have resulted in increases in the U and UH_2_ concentrations, irrespective of DPD activity (16). Finally, two patients with [UH_2_]:[U] < 1 (also corresponding to complete deficiency of DPD activity) did not carry defective alleles, as assessed using genotyping or NGS. Based on these results, *DPYD* allelic variants may be poor predictors of complete DPD deficiency, and the absence of a mutation does not exclude the possibility of complete DPD deficiency.

**Table 4:** Genotypes and phenotypes of individuals predicted to have complete DPD deficiency.

Uracil (U)	UH_2_	UH_2_/U	c.1679T>G	c.1905 + 1G>A	c.2846A>T	c.1129–5923C>G	Activity score
320	127	0.4	NM	HM	HM	NM	0.5
17	95	5.3	NM	HM	NM	HM	0.5
337	479	1.4	NM	NM	HM	NM	1
140	92	0.4	NM	NM	NM	NM	2
125	110	0.9	NM	NM	NM	NM	2

*HM* heterozygous, *NM* not mutated.

### Next-generation sequencing to identify rare genetic *DPYD* variants

*DPYD* gene sequencing was performed in 111 individuals with discordant genotype-phenotype correlations ([U] > 16 ng/ml or [UH_2_/][U] < 10 and *DPYD* wild-type genotype). Eighteen genetic variants were identified; all were missense mutations (Table S1) and the variant c.557A>G (p.Y186C; rs11523289) was the only variant to achieve a consensus regarding pathogenicity (activity score of 0.5, PharmGKB evidence level 1A for toxicity/adverse drug event (https://www.pharmgkb.org/clinicalAnnotation/1183703784)). Seven (7) patients (6%) were carriers of this variant and were predicted as having intermediate metabolism.^6^ Thus, in the subgroup of 111 patients with a genotype-phenotype discordance, whole-exome sequencing of the *DPYD* gene allowed us to correctly reclassify 6% (7/111) of the patients with DPD deficiency.

## Discussion

We describe here the largest series of pre-treatment DPD phenotyping combined with *DPYD* genotyping in non-selected patients with cancer. This timely resource is clinically relevant and consistent with the recent regulation in France related to the requirement for DPD phenotyping before the administration of fluoropyrimidines. The generalisation of a pre-treatment screen for DPD deficiency will exert a substantial effect on the volume of prescriptions of this test and will constitute a challenge in terms of health-care resources.^20^ Therefore, a comprehensive and accurate knowledge of the distribution of DPD activities and their relationships with *DPYD* mutations in a large and non-selected population is critical.

Since [U] and the [UH_2_]:[U] ratio are highly correlated with DPD activity in PBMC^1^ and *DPYD* variants show limitations to predict deficient DPD enzymatic activity as determined by measuring [U] and/or [UH_2_]:[U], we conclude that DPD phenotyping is a more appropriate approach to screen for DPD deficiency. However, our study provides information on the comparative performances of DPD phenotyping and *DPYD* genotyping in predicting fluoropyrimidine toxicity. Pre-treatment screening for the most frequently observed *DPYD* genetic variants and dose individualisation improve the safety of the patients,^10,25^ [U] and [UH_2_]:[U] might more comprehensively reflect DPD enzymatic activity than *DPYD* variants alone. A combined DPD phenotype-genotype testing to screen for DPD deficiency is a potentially clinically relevant approach,^15^ but it remains to be demonstrated that its yield a significantly higher predictive value.^26^ Hence, a majority of patients with DPD predicted to have intermediate or low metabolism according to activity scores based on the sum of alleles in fact have a normal DPD activity level based on [U] and [UH2]:[U] levels. This finding holds true for patients with partial DPD deficiency and complete DPD deficiency and may reflect a low penetrance of the mutations on the phenotype. However, a negative genetic test has a greater than 90% chance of an association with normal DPD activity.

Formal recommendations of DPD phenotype-guided dose individualisation of fluoropyrimidine therapy, similar to *DPYD* genotype-guided dose individualisation, should be elaborated and validated in prospective clinical studies. The clinical relevance of DPD phenotype screening will only be established in trials comparing DPD phenotype-guided and *DPYD* genotype-guided dose individualisation of fluoropyrimidines in patients with cancer. These studies might also help researchers define optimal thresholds for [U] and [UH_2_]:[U], which, unlike the results of genotyping tests, have the disadvantage of being continuous, with the risk of severe toxicity increasing proportionally with increasing values. The current [U] and [UH_2_]:[U] cut-off values used to define DPD deficiency likely overestimate the prevalence of an actual DPD deficiency, which might increase the risk to patients under the current dosing regimen. Thus, trials comparing current cut-off values and more stringent thresholds for phenotype-guided dose individualisation of fluoropyrimidine therapy would ultimately help clinicians to better tailor the fluoropyrimidine doses administered to patients with cancer.

Although *DPYD* mutations are poor predictors of DPD activity, they are better predictors of partial DPD deficiency when it is estimated by measuring the [UH_2_]:[U] ratio compared with [U] alone. This assumption is supported by the fact that [UH_2_]:[U] is the ratio of the product to the substrate of DPD, and therefore more accurately reflects the enzymatic activity of DPD than [U] alone. Consistent with this finding, several studies have shown a significant correlation between the [UH_2_]:[U] ratio and systemic fluorouracil exposure and the degree of toxicity.^23,24,27^ However, some debate exists regarding whether [UH_2_]:[U] or [U] is the more reliable predictor of the pharmacological response. While most available studies have correlated the [UH_2_]:[U] ratio to fluoropyrimidine-associated toxicity,^1,5,7,14,16^ some studies have shown that [U] better correlates with fluorouracil plasma clearance than [UH_2_]:[U],^14^ and consequently [U] may be superior to the [UH_2_]:[U] ratio for predicting toxicity.^7^

The complementary and additional information provided by the concomitant assessment of [U] and the [UH_2_]:[U] ratio remains to be examined in specific settings. For example, the rapid or ultra-rapid DPD phenotype is poorly described, and its clinical impact is likely underestimated.^28^ However, it could be considered a potent predictor of critical end-points, such as the response to the chemotherapy or patient survival. In this case, DPD phenotyping becomes critically relevant based on the findings of pharmacokinetic studies aiming to balance therapeutic effectiveness with safety that have proposed target ranges using the area under the curve (AUC) and that have shown that in phase III trials, > 65% of the patients are below the target AUC, less than 20% are above the target range, and only 15% of the patients have an AUC for the drug within the target range.^29^ The implementation of more sensitive tests for DPD deficiency, which appears to be the case for DPD phenotyping instead of *DPYD* genotyping, would theoretically expose a greater proportion of patients to the risk of being undertreated with fluoropyrimidine and, consequently, of being less responsive to the chemotherapy. Dose escalation after the first cycle to achieve maximal safe exposure is probably the most reasonable therapeutic strategy in these cases.

The sensitivity of *DPYD* genetic testing depends on the number of variants investigated, and a genetic analysis investigating only a selected panel of variants with decreased or no function will not exclude DPD defects due to the presence of rare genetic variants. Alternatively, investigating the complete coding sequence of *DPYD* would theoretically improve the predictive value of the screening test. The results of our NGS analysis do not reveal a substantial enrichment of rare *DPYD* variants in mutations predicted to cause functional alterations, since only one pathogenic variant (c.557A>G, p.Y186C) was identified in seven patients (6%), indicating that as a complementary approach to genotyping for the detection of rare mutations, NGS does not seem to increase the predictive performances of the genetic approach, at least in European populations. However, complementary genetic testing might help to confirm the genetic origin of the enzymatic deficiency, particularly in patients with complete DPD deficiency, and to justify family counselling.

A limitation of this study is the lack of clinical outcomes, including the rate of grade 3/4 toxicities. The retrospective design of the study did not allow us to evaluate the impact of DPD deficiency screening on the occurrence of toxicities during fluoropyrimidine treatment. These patients were followed in numerous oncology departments throughout France and we could not generate a data collection gathering biological and clinical variables of the patients screened. The primary end point of our study was to examine the relationship between DPD phenotypes and *DPYD* genotypes, and to what extent *DPYD* genotypes reflect pre-treatment DPD activity estimated by measuring [U] or [UH_2_]:[U] in a pre-treatment screening strategy. As such, our aim was not to compare the performances of two methods of DPD deficiency screening for fluoropyrimidines dose adjustment strategy, which would have required a prospective study to collect clinical outcomes. However, a clinical trial comparing toxicity rates after dose adjustment according to the results of genotyping or phenotyping testing to detect DPD deficiency will provide critical information on the best strategy.

In conclusion, common *DPYD* variants alone are moderately predictive of DPD deficiency and DPD phenotyping is considered the most appropriate method to screen for DPD deficiency. An assessment of [UH_2_]:[U] ratio can easily be implemented for routine screening because it rapidly provides reliable results, while allowing a more exhaustive identification of patients at risk of severe toxicity due to DPD deficiency. Further prospective randomised clinical studies should be performed to compare safety and efficacy of fluoroprymidines-based chemotherapy after dose adjustment according to [UH2]:[U] or [U] pretherapeutic determination.

## Supplementary information

Supplementary material

## Data Availability

Data supporting the results reported in the article can be obtained upon request to the corresponding author.

## References

[1] Meulendijks D, Henricks LM, Jacobs BAW, Aliev A, Deenen MJ, de Vries N (2017). Pretreatment serum uracil concentration as a predictor of severe and fatal fluoropyrimidine-associated toxicity. Br. J. Cancer.

[2] Andre T, Colin P, Louvet C, Gamelin E, Bouche O, Achille E (2003). Semimonthly versus monthly regimen of fluorouracil and leucovorin administered for 24 or 36 weeks as adjuvant therapy in stage II and III colon cancer: results of a randomized trial. J. Clin. Oncol..

[3] Mikhail SE, Sun JF, Marshall JL (2010). Safety of capecitabine: a review. Expert Opin. Drug Saf..

[4] Barin-Le Guellec C, Lafay-Chebassier C, Ingrand I, Tournamille JF, Boudet A, Lanoue MC (2020). Toxicities associated with chemotherapy regimens containing a fluoropyrimidine: a real-life evaluation in France. Eur. J. Cancer.

[5] van Kuilenburg AB (2004). Dihydropyrimidine dehydrogenase and the efficacy and toxicity of 5-fluorouracil. Eur. J. Cancer.

[6] Amstutz U, Henricks LM, Offer SM, Barbarino J, Schellens JHM, Swen JJ (2018). Clinical Pharmacogenetics Implementation Consortium (CPIC) guideline for dihydropyrimidine dehydrogenase genotype and fluoropyrimidine dosing: 2017 update. Clin. Pharm. Ther..

[7] Etienne-Grimaldi MC, Boyer JC, Beroud C, Mbatchi L, van Kuilenburg A, Bobin-Dubigeon C (2017). New advances in DPYD genotype and risk of severe toxicity under capecitabine. PLoS ONE.

[8] Toffoli G, Giodini L, Buonadonna A, Berretta M, De Paoli A, Scalone S (2015). Clinical validity of a DPYD-based pharmacogenetic test to predict severe toxicity to fluoropyrimidines. Int J. Cancer.

[9] Terrazzino S, Cargnin S, Del Re M, Danesi R, Canonico PL, Genazzani AA (2013). DPYD IVS14+1G>A and 2846A>T genotyping for the prediction of severe fluoropyrimidine-related toxicity: a meta-analysis. Pharmacogenomics.

[10] Henricks LM, Lunenburg C, de Man FM, Meulendijks D, Frederix GWJ, Kienhuis E (2018). DPYD genotype-guided dose individualisation of fluoropyrimidine therapy in patients with cancer: a prospective safety analysis. Lancet Oncol..

[11] Meulendijks D, Henricks LM, Sonke GS, Deenen MJ, Froehlich TK, Amstutz U (2015). Clinical relevance of DPYD variants c.1679T>G, c.1236G>A/HapB3, and c.1601G>A as predictors of severe fluoropyrimidine-associated toxicity: a systematic review and meta-analysis of individual patient data. Lancet Oncol..

[12] Swen JJ, Nijenhuis M, de Boer A, Grandia L, Maitland-van der Zee AH, Mulder H (2011). Pharmacogenetics: from bench to byte–an update of guidelines. Clin. Pharm. Ther..

[13] Sistonen J, Buchel B, Froehlich TK, Kummer D, Fontana S, Joerger M (2014). Predicting 5-fluorouracil toxicity: DPD genotype and 5,6-dihydrouracil:uracil ratio. Pharmacogenomics.

[14] Boisdron-Celle M, Remaud G, Traore S, Poirier AL, Gamelin L, Morel A (2007). 5-Fluorouracil-related severe toxicity: a comparison of different methods for the pretherapeutic detection of dihydropyrimidine dehydrogenase deficiency. Cancer Lett..

[15] Boisdron-Celle M, Capitain O, Faroux R, Borg C, Metges JP, Galais MP (2017). Prevention of 5-fluorouracil-induced early severe toxicity by pre-therapeutic dihydropyrimidine dehydrogenase deficiency screening: Assessment of a multiparametric approach. Semin Oncol..

[16] van Kuilenburg AB, Haasjes J, Richel DJ, Zoetekouw L, Van Lenthe H, De Abreu RA (2000). Clinical implications of dihydropyrimidine dehydrogenase (DPD) deficiency in patients with severe 5-fluorouracil-associated toxicity: identification of new mutations in the DPD gene. Clin. Cancer Res..

[17] Launay M, Ciccolini J, Fournel C, Blanquicett C, Dupuis C, Fakhry N (2017). Upfront Dpd deficiency detection to secure 5-Fu administration: part 2- application to head-and-neck cancer patients. Clin. Cancer Drugs.

[18] Launay M, Dahan L, Duval M, Rodallec A, Milano G, Duluc M (2016). Beating the odds: efficacy and toxicity of dihydropyrimidine dehydrogenase-driven adaptive dosing of 5-FU in patients with digestive cancer. Br. J. Clin. Pharm..

[19] Yang CG, Ciccolini J, Blesius A, Dahan L, Bagarry-Liegey D, Brunet C (2011). DPD-based adaptive dosing of 5-FU in patients with head and neck cancer: impact on treatment efficacy and toxicity. Cancer Chemother. Pharm..

[20] Jacobs BA, Rosing H, de Vries N, Meulendijks D, Henricks LM, Schellens JH (2016). Development and validation of a rapid and sensitive UPLC-MS/MS method for determination of uracil and dihydrouracil in human plasma. J. Pharm. Biomed. Anal..

[21] Coudore F, Roche D, Lefeuvre S, Faussot D, Billaud EM, Loriot MA (2012). Validation of an ultra-high performance liquid chromatography tandem mass spectrometric method for quantifying uracil and 5,6-dihydrouracil in human plasma. J. Chromatogr. Sci..

[22] Loriot, M. A., Ciccolini, J., Thomas, F., Barin-Le-Guellec, C., Royer, B., Milano, G. et al. [Dihydropyrimidine dehydrogenase (DPD) deficiency screening and securing of fluoropyrimidine-based chemotherapies: update and recommendations of the French GPCO-Unicancer and RNPGx networks]. *Bull. Cancer*. **105**, 397–407 (2018) . 10.1016/j.bulcan.2018.02.001 29486921

[23] Gamelin E, Boisdron-Celle M, Guerin-Meyer V, Delva R, Lortholary A, Genevieve F (1999). Correlation between uracil and dihydrouracil plasma ratio, fluorouracil (5-FU) pharmacokinetic parameters, and tolerance in patients with advanced colorectal cancer: A potential interest for predicting 5-FU toxicity and determining optimal 5-FU dosage. J. Clin. Oncol..

[24] Neto OV, Raymundo S, Franzoi MA, do Carmo Artmann A, Tegner M, Muller VV (2018). DPD functional tests in plasma, fresh saliva and dried saliva samples as predictors of 5-fluorouracil exposure and occurrence of drug-related severe toxicity. Clin. Biochem..

[25] Meulendijks D, Cats A, Beijnen JH, Schellens JH (2016). Improving safety of fluoropyrimidine chemotherapy by individualizing treatment based on dihydropyrimidine dehydrogenase activity—ready for clinical practice?. Cancer Treat. Rev..

[26] Etienne-Grimaldi MC, Le Guellec CB, Boyer JC, Chatelut E, Evrard A, Loriot MA (2017). Prevention of 5-fluorouracil-induced early severe toxicity by pre-therapeutic dihydropyrimidine dehydrogenase deficiency screening: The multiparametric approach is not convincing. Semin Oncol..

[27] Galarza AF, Linden R, Antunes MV, Hahn RZ, Raymundo S, da Silva AC (2016). Endogenous plasma and salivary uracil to dihydrouracil ratios and DPYD genotyping as predictors of severe fluoropyrimidine toxicity in patients with gastrointestinal malignancies. Clin. Biochem..

[28] Botticelli A, Borro M, Onesti CE, Strigari L, Gentile G, Cerbelli B (2016). Degradation rate of 5-Fluorouracil in metastatic colorectal cancer: a new predictive outcome biomarker?. PLoS ONE.

[29] Saam J, Critchfield GC, Hamilton SA, Roa BB, Wenstrup RJ, Kaldate RR (2011). Body surface area-based dosing of 5-fluoruracil results in extensive interindividual variability in 5-fluorouracil exposure in colorectal cancer patients on FOLFOX regimens. Clin. Colorectal Cancer.

