# Supplementary material for: A comprehensive population-based study comparing the phenotype and genotype in a pretherapeutic screen of dihydropyrimidine dehydrogenase deficiency
Source: Br J Cancer. 2020 Jun 29;123(5):811–8. doi: 10.1038/s41416-020-0962-z (PMC7462856; doi:10.1038/s41416-020-0962-z)
Supplement: Supplementary file 1 — Supplementary material [file 41416_2020_962_MOESM1_ESM.pdf]

Figure S1

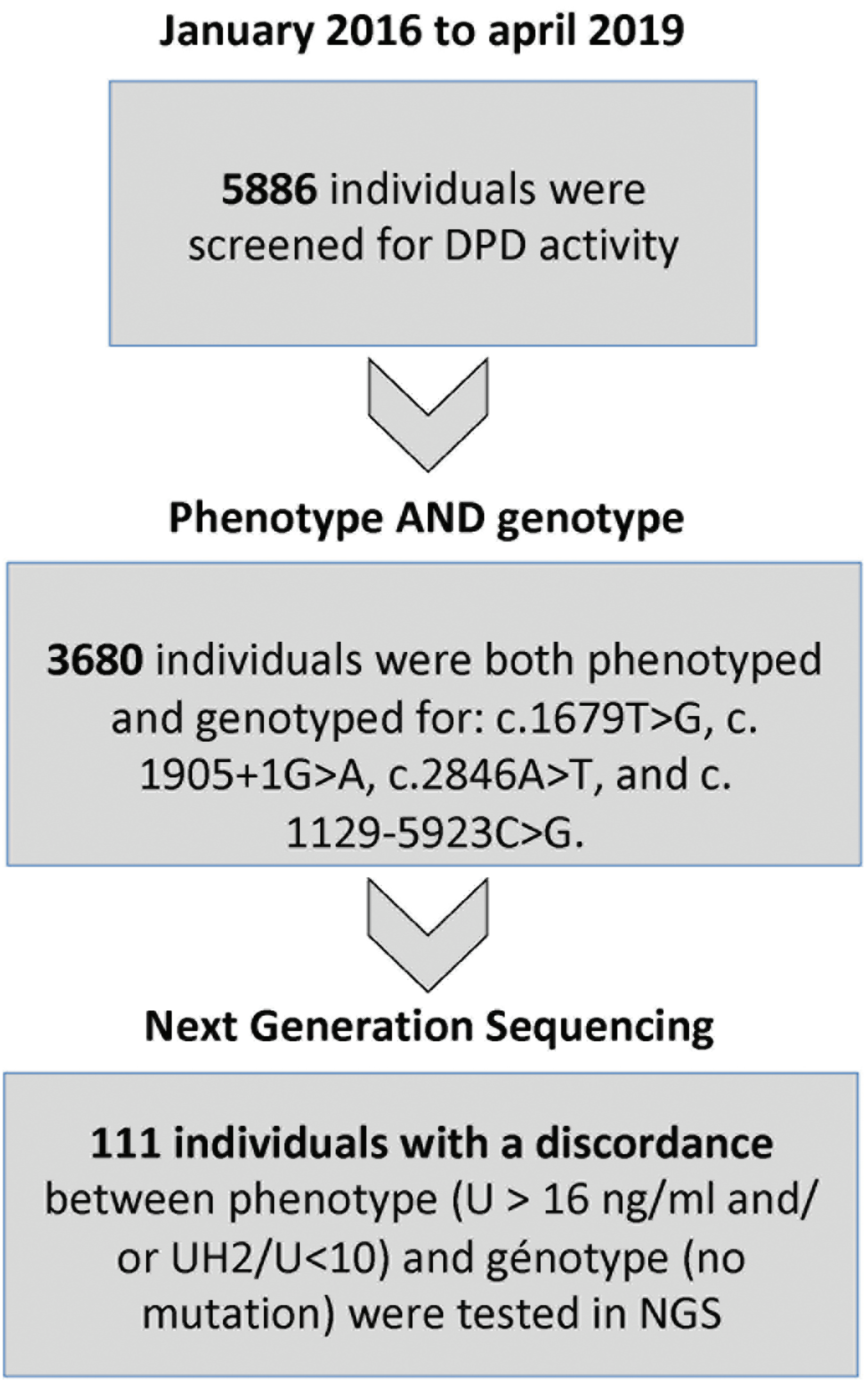
January 2016 to april 2019
5886 individuals were screened for DPD activity
Phenotype AND genotype
3680 individuals were both phenotyped and genotyped for: c.1679T>G, c. 1905+1G>A, c.2846A>T, and c. 1129-5923C>G.
Next Generation Sequencing
111 individuals with a discordance between phenotype (U > 16 ng/ml and/ or UH2/U<10) and génotype (no mutation) were tested in NGS

Figure S2

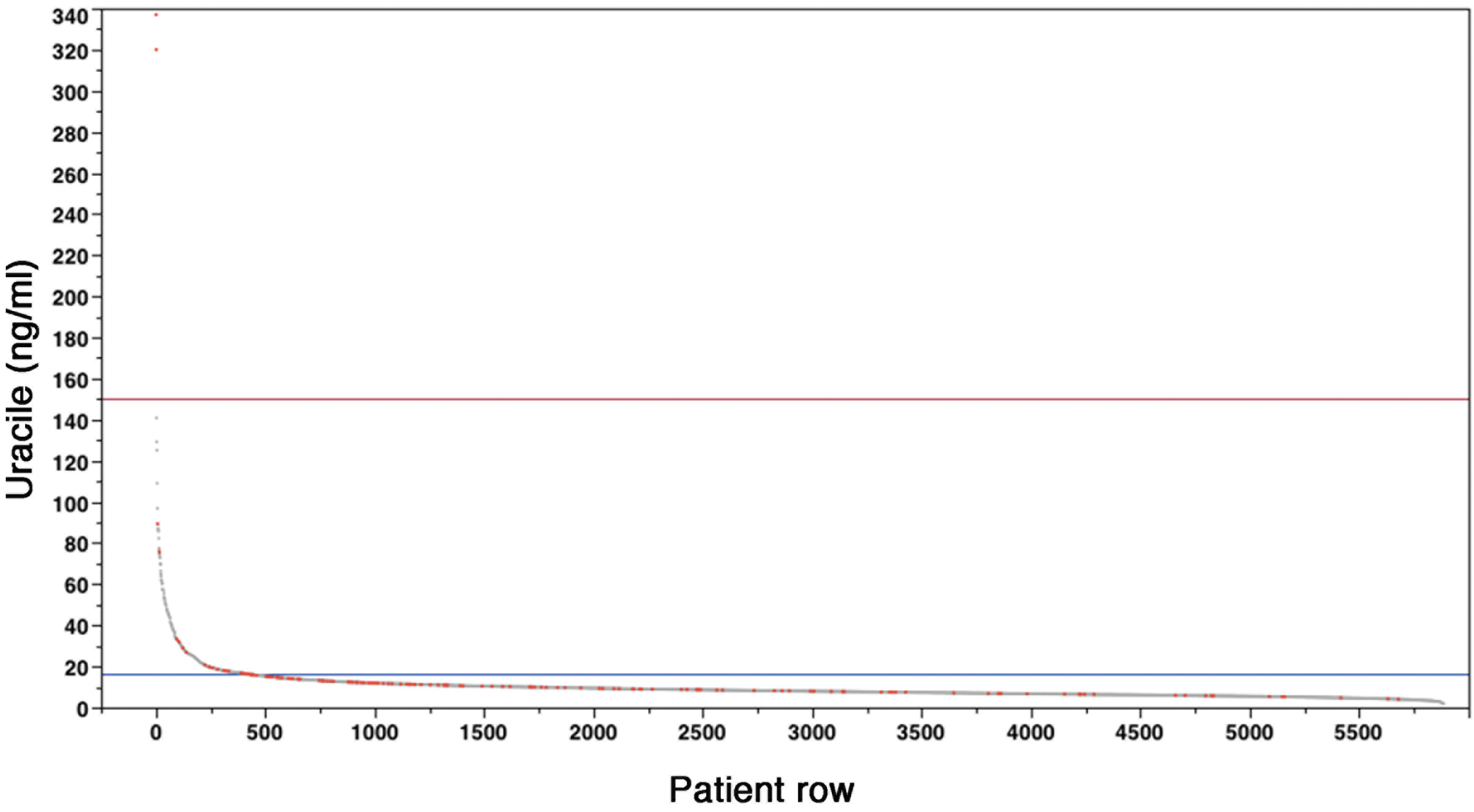

340
320
300
280
260
240
220
200
180
160
140
120
100
80
60
40
20
0
Uracile (ng/ml)
0
500
1000
1500
2000
2500
3000
3500
4000
4500
5000
5500
Patient row

Figure S3

A

Uracile

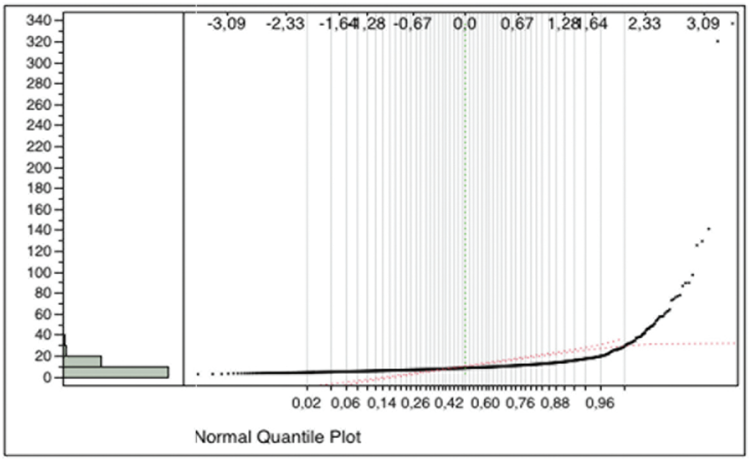

B

UH2:U

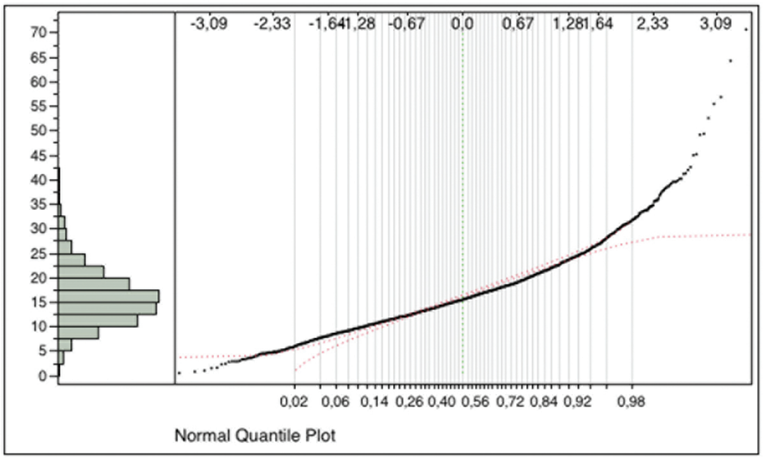

Figure S4

A

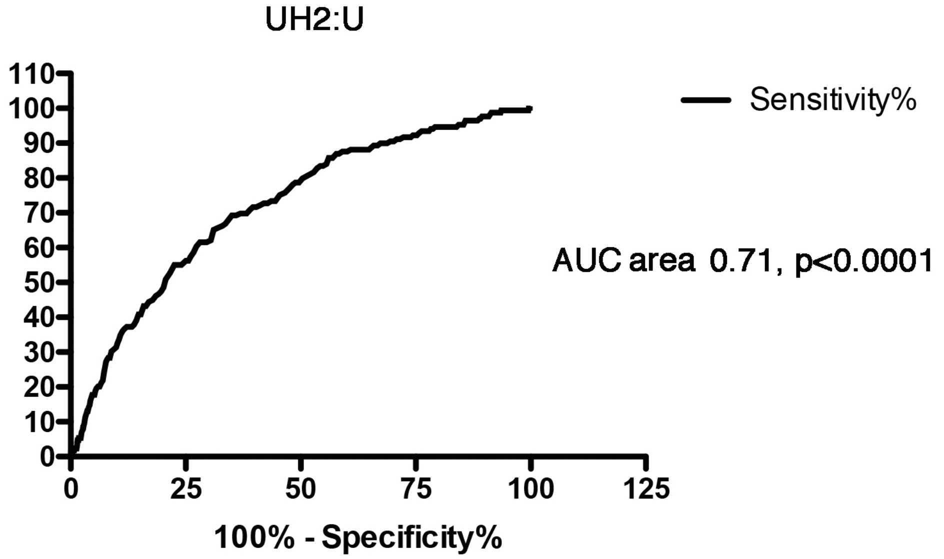
UH2:U
Sensitivity%
AUC area 0.71, p<0.0001
100% - Specificity%
0
25
50
75
100
125
0
10
20
30
40
50
60
70
80
90
100
110

B

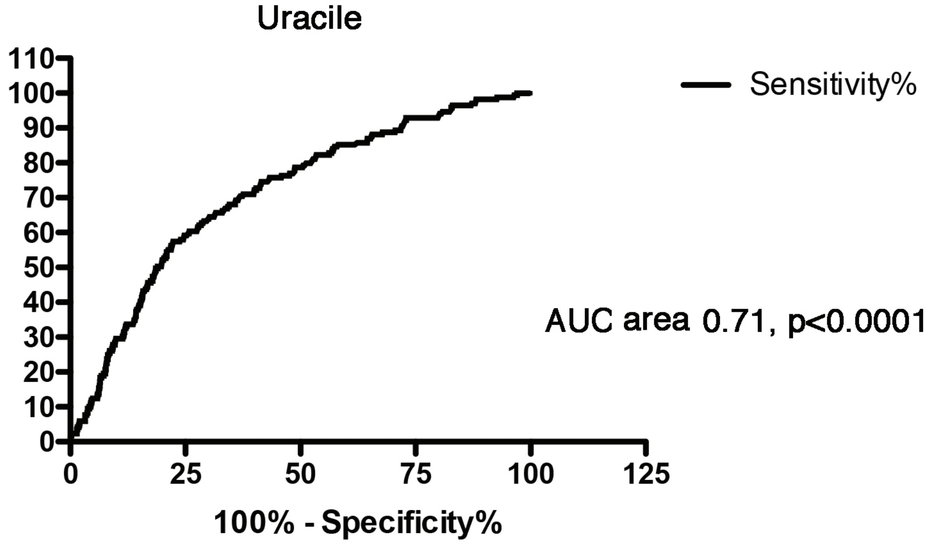
Uracile
Sensitivity%
AUC area 0.71, p<0.0001
100% - Specificity%
0
25
50
75
100
125
0
10
20
30
40
50
60
70
80
90
100
110

Figure S1. Flow chart of the study population.

Figure S2. Repartition of [U] values of the 5886 patients with cancer. Gray dots represent each individual with the corresponding [U] value and pink dots represent individuals with a defective *DPYD* allelic variant.

Figure S3. Distribution of [U] and [UH_2]:[U]. Normal quantile plots were created to visualize the extent to which the variable was normally distributed. If a variable is normally distributed, the normal quantile plot approximates a diagonal straight line.

Figure S4. Receiver operating characteristic curves of the performances of [U] and [UH_2]:[U] to predict the mutation status of *DPYD*.

Table S1. Mutational spectrum of 111 patients with a DPD deficiency without none of the 4 most common *DPYD* defective allelic variant detected in single analysis

Haplotype	rsID	Nucleotide change	Protein change	CPIC	SIFT	N of carriers
*9A	rs1801265	c.85T>C	p.C29R	Normal	Deleterious	94
*5	rs1801159	c.1627A>G	p.I543V	Normal	Deleterious	44
	rs2297595	c.496A>G	p.M166V	Normal	Deleterious	33
*6	rs1801160	c.2194G>A	p.V732I	Normal	Tolerated	23
*4	rs1801158	c.1601G>A	p.S534N	Normal	Deleterious	2
	rs17376848	c.1896T>C	p.F632F	Normal	-	4
	rs115232898	c.557A>G	p.Y186C	Decreased	Deleterious	7
	rs114096998	c.3067C>A	p.P1023T	Normal	Deleterious	3
	rs57918000	c.1371C>T	p.N457N	Normal	-	1
	rs61622928	c.1218G>A	p.M406I	Normal	Tolerated	1
	rs189768576	c.220C>T	p.Arg74Ter	-	-	1
*9B	rs1801267. rs1801265	c.2657G>A. c.85T>C	p.R886H. p.C29R	Normal	Deleterious	1
	rs146170505	c.661G>T	p.E221Ter	-	-	1
	rs141376128	c.975C>T	p.H523H	Normal	Tolerated	1
	rs72549305	c.1108A>G	p.I370V	Normal	Tolerated	1
	rs45589337	c.775A>G	p.K259E	Normal	Deleterious	1
		c.1863G>T	p.W621C	-	Deleterious	1
		c.328T>G	p.Y110D	-	Deleterious	1
